# Drug prescribing patterns at primary health care level and related out-of-pocket expenditures in Tajikistan

**DOI:** 10.1186/s12913-016-1799-2

**Published:** 2016-10-06

**Authors:** Morgane Donadel, Gulzira Karimova, Ruslan Nabiev, Kaspar Wyss

**Affiliations:** 1 Swiss Centre for International Health, Swiss Tropical and Public Health Institute, Socinstr 57, Basel, 4002 Switzerland; 2 Department of Tropical Medicine and Clinical International Health, Centre Hospitalier Universitaire de Bordeaux, Place Amélie Raba-Léon, 33076 Bordeaux, Cedex France; 3 Enhancing Primary Health Care Services Project, Shota Rustaveli, 35 Dushanbe, Tajikistan; 4 Centre of Sociological Research “Zerkalo”, 7 Rudaki Avenue, Dushanbe, Tajikistan; 5 University of Basel, Basel, Switzerland

**Keywords:** Tajikistan, Drug prescribing patterns, Out-of-pocket expenditure, Primary health care, Health financing

## Abstract

**Background:**

The Government of Tajikistan is reforming its health system to make access more equitable. Nonetheless, out-of-pocket expenditures (OPE) remain a key modality for purchasing health care. Drugs remain a major driver of household expenditures for health. We conducted a household survey to investigate drug prescribing patterns at primary health care (PHC) level as well as the related OPE.

**Methods:**

Adult patients in eight districts who had visited a PHC facility in the period March to May 2014 were interviewed at home, using a structured questionnaire. A descriptive analysis was conducted and regression models were constructed to identify factors influencing the number of drugs provided and the types of drugs prescribed.

**Results:**

There were 1281 (80.1 %) patients who received a drug prescription after visiting a doctor at PHC level. 16.2 % of them had five or more drugs prescribed concomitantly. The number of drugs prescribed to patients ranged from 0 to 8 and was statistically different across regions (RRS region =3.3; Khatlon region = 3.1; *p* = 0.05), after adjusting for age and sex. In 31.1 % of cases, prescriptions included an intra-venous (IV) injection; in 45.6 % of cases, a non-IV injection; in 52.9 % of cases, an antibiotic; and in 61.0 % of cases, vitamins. Patients suffering from a respiratory disease had higher odds of being prescribed an IV injection and antibiotics. Vitamins were widely prescribed across all diseases. In 94.5 % of cases, the patients interviewed procured at least one of the prescribed drugs. Among those who received a prescription, 2.0 % were not able to procure at least one drug due to a lack of money. In 94.9 % of cases, respondents reported purchasing drugs in private pharmacies. Median expenditures for drugs procured following consultation were 45 TS (US$ 6.9) corresponding to 77.6 % of total expenditures related to the visit (58 TS, US$ 8.8).

**Conclusions:**

In a context where OPE are important, drugs represent an important income source for health service providers. Such a situation does not favour rational prescribing nor efficient service delivery, and is potentially harmful for patients. In particular, the economic ramifications cause high levels of expenditure for patients and households with detrimental, knock-on effects in the more vulnerable segments of the population.

**Electronic supplementary material:**

The online version of this article (doi:10.1186/s12913-016-1799-2) contains supplementary material, which is available to authorized users.

## Background

Tajikistan is a landlocked and mountainous country in Central Asia with approximately 8.3 million inhabitants, most of whom (73.3 %) live in rural areas [1]. It is classified as a lower middle income country with a Gross Domestic Product (GDP) per capita of US$ 2655 (in current international dollar, purchasing power parity) in 2014 and a GDP growth of 6.7 % in 2014 [1]. Tajikistan faces a double burden of both high communicable and non-communicable disease rates. Anaemia and malnutrition are other pressing health concerns.

After the collapse of the Soviet Union in 1991, and Tajikistan’s independence and subsequent civil war, a dramatic decrease of government health expenditure occurred, which triggered the system’s dependence on private out-of-pocket payments, including informal, under-the-table payments [2]. However, total health expenditure remains low and was estimated to amount to 6.8 % in 2013 [3], although this was still a higher share of GDP devoted to health in 2013 than in any of the other – and wealthier – Central Asian states (Kazakhstan, Kyrgyzstan, Turkmenistan and Uzbekistan). Given its low GDP per capita, Tajikistan had the lowest per capita spending on health in the WHO European Region in 2013, amounting to only USD 170 (current international dollar, purchasing power parity) [4], and the country’s health system remains underfunded. Previously focusing on secondary and tertiary health care, since 2002 the government has shown commitment to foster the role of primary health care (PHC), although the share of government funds devoted to PHC, at 34.8 % in 2013 [5], remains comparatively low. Being governmental employees, family doctors working at PHC level earn low wages. They were estimated to range between US$ 123 and US$ 153 per month in 2013 such that workers often rely on informal payments and in-kind contributions to earn additional income [5].

Private out-of-pocket payments are nowadays the main source of health financing in Tajikistan, accounting for 60.1 % of total health expenditure in 2013 [3]. Out-of-pocket expenditure (OPE) for health is defined by the World Health Organization as “any direct outlay by households, including gratuities and in-kind payments, to health practitioners and suppliers of pharmaceuticals, therapeutic appliances, and other goods and services whose primary intent is to contribute to the restoration or enhancement of the health status of individuals or population groups” [3]. Already in 2003, Falkingham reported that “out-of-pocket payments deter people from seeking medical assistance and once advice has been sought, from receiving the most appropriate treatment” [6]. According to a survey conducted in 2011, 18.8 % of households faced catastrophic expenditure on health (defined as out-of-pocket spending that exceeds 40 % of a households non-subsistence spending), with this share rising to 26.7 % in the lowest income quintile [7].

Public funding for the purchase of medicines has declined significantly since 1991, but the cost of pharmaceuticals, most of which are imported, has increased substantially. In 2013, only 2.8 % of government expenditure on health was spent on pharmaceuticals, in both inpatient and outpatient care [5]. This contrasts with 13 % of total (mostly public) health expenditure being spent on pharmaceuticals in 1991 [4]. The largest share of household OPE for health is dedicated to pharmaceuticals [2].

While in many parts of the world various studies have described and analysed drug prescribing to patients and the purchasing of medicines, including prescription of essential drugs through primary care practitioners, not much is known about this issue in the Central Asian context. One of the few studies that have been undertaken so far examined prescribing practices of doctors in rural PHC clinics in Uzbekistan and found “a very high use of injections, a low rate of generic drugs prescribed, a high use of antibiotics, and a high rate of polypharmacy” [8].

In order to analyse the situation in Tajikistan, a household survey was conducted in 2014 to investigate drug prescribing and purchasing patterns among patients who visited a PHC facility. This survey was the follow-up of two rounds of earlier surveys that aimed to capture the scale and determinants of patients’ OPE when using PHC services [9, 10]. The study was conducted within the Enhancing Primary Health Care Services project -Tajikistan, a bilateral health service development project funded by the Swiss Agency for Cooperation and Development, aiming at improved population health and enhanced access to health services.

## Methods

### Questionnaire, study population and sampling strategy

A cross-sectional and community-based study was carried out among patients who had visited a PHC facility in the period March to May 2014. A structured questionnaire (Additional file 1) was used to ask patients about their experience with PHC services and with the prescription and purchase of drugs during their most recent visit of a governmental health service provider. Questions on total OPE and demographics were also included in the questionnaire.

The study covered eight predominantly rural districts in Tajikistan. Dangara, Hamadoni and Vose districts are part of the Khatlon region while Faizabad, Rudaki, Shakrinav, Tursunzade are part of the Region of Republican Subordination (RRS). A multi-level sampling strategy was used to select the study population representing the respective geographic location. The number of PHC facilities that were included in the study was determined proportionally to the total number of PHC facilities within a given district. Their geographical accessibility was then taken into account, with 50 % of facilities included in the survey classified as remote and 50 % as more accessible, based on the distance to the district capital. The second stage concerned the selection of patients themselves. In each district, the number of patients to include was established proportionally to the total number of adult patients a particular facility had received in the previous year, as captured by the national health information system. Thus, the number of respondents from each facility was proportional to the total number of patients reported to have visited that facility in the previous year.

Facility registries of PHC providers were then used to select patients randomly. All included health service providers were staffed with at least one doctor, either a family doctor (FD) or a specialist.

The sample size requirement was based on the premises to detect at least a 10 % difference in total out-of-pocket payments and a 7 % difference in access to medicine across three socioeconomic groups using a power of 90 % and a significance level of 5 %. It resulted in a required sample of 750 patients. It was then decided to round up to 800 patients and we further doubled the sample size to 1600 patients so as to be able to stratify along the four districts which had been covered in the previous surveys and along those four newly included ones.

### Data collection and analysis

Patients were visited at home and interviewed by trained interviewers. Information on drug prescription and procurement following their last visit to a PHC facility was based on their memory and, if available, the prescription.

Data were collected and entered using tablets in June 2014 by Zerkalo, Centre for Sociological Research. In addition, there was an independent monitor in charge of quality assurance in order to ensure that the outlined methodology was respected.

Data were analysed using Stata 13 software. Qualitative variables were described by absolute value, percentage and 95 % confidence interval based on binomial law. Quantitative variables were described by their absolute value, the mean, and a 95 % confidence interval of the mean, a standard deviation, median and range. Regression models were constructed to identify factors influencing the number of drugs provided to patients and the types of drugs prescribed. Results were age- and gender adjusted, to allow comparison across these demographic variables.

## Results

### Patient characteristics

In the eight study districts, 1611 patients were randomly selected to be followed up at home and interviewed. However, 446 (27.7 %) of them could not be interviewed at home after three attempts and were replaced by another eligible patient who had previously visited a PHC facility. Finally, 1599 adult patients were included in the analysis; 18.6 % of respondents were male and 81.4 % were female (Table 1). The median age of respondents was 30 years, the median number of years of education was 10 and the median number of visits to a PHC service provider in the past 12 months was 4.

**Table 1:** Patients’ characteristics

Variable		Number	Percent
Number of respondents		1599	100
Gender	Female	1302	81.4
	Male	297	18.6
Median Age		30	
Median number of years of education		10	
Region	RRS	575	36.0
	Khatlon	1024	64.0
Median number of visits to a FD in the past 12 months		4	
Reasons to consult	Respiratory diseases	418	26.1
	Cardiovascular diseases	193	12.1
	Pregnancy	274	17.1
	Gastrointestinal diseases	156	9.8
	Diarrhoea	140	8.8
	Genitourinary diseases	132	8.2
	Diabetes	19	1.2
	Anaemia	83	5.2
	Injuries	43	2.7
	Skin disease	22	1.4
	Other	119	7.4

In 26.1 % of cases, visits to PHC services were due to respiratory diseases, 17.1 % were related to pregnancy, 12.1 % to cardiovascular diseases, 9.8 % to gastrointestinal diseases, 8.8 % to diarrhoea, 8.2 % to genitourinary diseases, 5.2 % to anaemia and 1.2 % to diabetes.

### Drug prescription

The vast majority of respondents (1281 patients, equivalent to 80.1 % of the sample) reported having received a drug prescription during their visit to a PHC provider (Table 2). The median number of drugs prescribed by the FD was 3, ranging from 1 to 8 or more, the first and third quartiles attaining 2 and 4 respectively (Table 2). The median number of drugs procured by patients was 3, with a range and quartiles equivalent to the number of drugs prescribed. 259 (16.2 %) patients were prescribed five or more drugs concomitantly, meeting the generally accepted threshold for polypharmacy [11] (Table 2). Although the difference was not statistically significant, women more often received antibiotics (53.8 % vs 49.0 %) and vitamins (62.5 % vs 54.4 %) after consulting their FD. The older the patient, the higher the number of drugs prescribed (3.0 among those 18 and 35 years old; 3.5 among those 36 and 55 years old; 3.9 among those older than 55; *p* < 10-3). Along this, the likelihood for an antibiotic injection increased. Regional disparities were observed, with patients living in Khatlon region receiving fewer drugs, after adjustments for age and gender (3.3 in the RRS vs 3.1 in the Khatlon region *p* < 0.05) (Table 3).

**Table 2:** Drug prescription during consultation

Variable	Number	Percent
Drug prescription	1281	80.1
Median number of drugs prescribed	3	
Prescription with > = 5 drugs	259	16.2

**Table 3:** Drug prescribing by gender, age and region (*N* = 1281)

Variable	Gender		Age			Region	
	Female	Male	18-35	36-55	>55	RRS	Khatlon
*N* (%)	1042 (81.3)	239 (18.7)	828 (64.6)	330 (25.8)	123 (9.6)	439 (34.3)	842 (65.7)
Mean number of drugs^*^	3.2	3.3	3.0	3.5	3.9	3.3	3.1
*SD*	1.5	1.4	1.4	1.5	1.7	1.5	1.4
% IV injections	31.4	29.7	23.4	37.9	64.2	37.2	19.4
% non IV injections	45.9	44.4	40.2	55.2	56.1	50.2	36.7
% Antibiotics	53.8	49.0	48.7	61.8	57.7	56.7	45.8
% Vitamins	62.5	54.4	62.0	59.1	59.4	62.2	58.5

^*^ The differences are significant across age groups (*P* < 10^-3^) and across regions (*P* < 0.05) after age and gender adjustments

### Characteristics of the drugs prescribed

When a prescription was issued (*N* = 1281), it included an intravenous injection in 31.1 % of cases; a non-intravenous (intradermal or intramuscular) injection in 45.6 % of cases and an antibiotic in 52.9 % of cases. Vitamins were issued in 61.0 % of cases (Table 4). The number of drugs prescribed was lowest when consulting for diabetes (mean of 2.7 and SD = 1.8) and highest when consulting for injuries (mean of 3.9 and SD = 2.0) (Table 4). Diabetics were the most likely to receive a prescription of an intravenous injection (60.0 %) while patients consulting for a genitourinary disease (supposedly infections) were most likely to be prescribed an antibiotics (64.4 %). Suffering from a respiratory disease increased the odds of being prescribed an antibiotic, after age and gender were adjusted for. Finally, vitamins and non-intravenous drugs were widely prescribed across all diseases (Table 5).

**Table 4:** Drug prescribing by reason to consult (*N* = 1281)

Variable	Reason to consult											
	All	Respiratory	Digestive	Cardiovascular	Diarrhoea	Pregnancy	Genitourinary	Injuries	Skin disease	Diabetes	Anaemia	Other
N	1281	324	115	156	114	220	101	38	22	15	74	102
Mean number of drugs	3.2	3.1	3.2	3.6	3.1	2.9	3.8	3.9	3.1	2.7	3.1	3.5
SD	1.5	1.3	1.5	1.7	1.2	1.3	1.6	2.0	1.2	1.8	1.4	1.6
% IV injections	31.1	17.9	28.7	50.0	29.0	26.8	47.5	42.1	4.6	60.0	40.5	32.4
% non IV injections	45.6	44.1	44.4	48.1	36.8	42.7	65.4	57.9	22.7	46.7	50.0	41.2
% Antibiotics	52.9	66.7	50.4	49.4	47.4	40.9	64.4	60.5	31.8	53.3	51.4	41.2
% Vitamins	61.0	63.6	64.4	55.8	60.5	66.8	57.4	44.7	54.6	40.0	68.9	52.9

**Table 5:** Multivariate analysis showing variables influencing the odds of buying different types of drugs (*N* = 1281)

Explanatory variable		Intravenous injection		Non-intravenous injection		Antibiotics		Vitamins	
		Coefficient (95 % CI)	*P*-value	Coefficient (95 % CI)	*P*-value	Coefficient (95 % CI)	*P*-value	Coefficient (95 % CI)	*P*-value
Age		-0.009 (-0.01- -0.007)	0.000	-0.006 (-0.008- -0.004)	0.000	-0.004 (-0.007- -0.001)	0.002		0.874
Gender		-0.12 (-0.19- -0.05)	0.000		0.066		0.052		0.103
Reason to consult	Respiratory	1		1		1		1	
	Digestive	-0.11(-0.21- -0.008)	0.034		0.799	0.22 (0.09-0.34)	0.001		0.998
	Cardiovascular	-0.21(-0.30- -0.11)	0.000		0.378	0.23 (0.11-0.35)	0.000		0.177
	Diarrhoea	-0.12(-0.22- -0.025)	0.014		0.235	0.22 (0.09-0.34)	0.001		0.411
	Pregnancy	-0.13(-0.21- -0.046)	0.002		0.914	0.25 (0.14-0.35)	0.000		0.89
	Genitourinary	-0.26(-0.36- -0.16)	0.000	-0.18 (-0.29- -0.064)	0.002		0.21		0.113
	Injuries	-0.16(-0.31- 0.00)	0.050		0.339		0.517	0.21 (0.026-0.40)	0.026
	Skin disease		0.097	0.23 (0.005-0.45)	0.045	0.38 (0.12-0.63)	0.004		0.376
	Diabetes	-0.25(-0.26- -0.03)	0.048		0.478		0.266		0.164
	Anaemia	-0.15(-0.26- -0.03)	0.013		0.849	0.19 (0.04-0.34)	0.011		0.958
	Other		0.085		0.241	0.32 (0.19-0.46)	0.000	0.13 (0.009-0.26)	0.036

### Drug procurement

Of the total number of patients who had received a prescription, 94.5 % were able to obtain at least part of the drugs while the remaining 5.5 % were unable to obtain their full prescription. Those who were not able to get drugs (*N* = 71) indicated that a lack of money was the most important reason (35.2 % of cases). Some patients did not feel the need to buy one or more of the prescribed drugs (23.9 % of cases). In 9.9 % of cases, patients declared not obtaining drugs due to a pharmacy being difficult to access geographically. It is noteworthy that at the pharmacy level, difficulties within the distribution chain also became apparent, as 7.0 % of patients reported stock-outs of a given drug in the 3 months assessment period (Table 6).

**Table 6:** Drug procurement

Variable		Number	Percent
Drugs obtained after being prescribed (*N* = 1281)		1210	94.5
Reasons for not obtaining (*N* = 71)	No pharmacy near by	7	9.9
	Lack of money	25	35.2
	Medicine not in stock	5	7.0
	No felt need	17	23.9
	Other	17	23.9

Private pharmacies are the most common place for drug procurement: 94.9 % of patients who procured at least one drug did so from a private vendor. In some few instances, the drugs were procured at a hospital (1.2 % of interviewees with at least one drug prescribed). The remaining patients reported obtaining drugs from the market (1.2 %) or free of charge from a PHC provider (1.5 %).

### Expenditures for drugs

The visit to a governmental PHC provider was related to total median expenditures of 58 TS (US$ 8.8) (mean expenditures of 121 TS; US$ 18.5; SD = 259) which included expenditures for admission, transportation and drugs. These costs were substantial and corresponded to a relevant share of household expenditures.

The median amount of money spent on drugs amounted to 45 TS (US$ 6.9) (mean expenditures of 98 TS; US$ 15.0; SD = 223), corresponding to 77.6 %, respectively more than three-quarters of the total household expenditure made in the course of a visit to a PHC provider.

## Discussion

### Limitations

More than 22 % of the patients initially selected through our sampling strategy were not interviewed, as most of them could not be found at home. Hypothetically, these 458 patients could have had systematically different answers from the ones who were interviewed, introducing selection bias. Although our findings on prescribing patterns are in line with previous research conducted in Central Asia, the precise figures need therefore to be interpreted with caution. Additionally, our sample over-represented females compared to males. Three reasons were identified to explain this situation: (1) in rural areas of Tajikistan, many males are working as labour migrants in Russia so that the demographic weight of women in the study regions is substantially higher; (2) PHC services are typically used by women and children so that their representation in the sample is expected to be higher and (3) as men were more frequently working and not at home at the time of the interview, they were in some instances replaced by a female patient. Furthermore and as the main focus of the study was to have representative information on patients’ OPE and on drug prescribing, the study was not assessing whether patients consulted for the first time or in the frame of a follow-up visit. However, the nature of the visit (first visit or follow-up) may influence the size of expenditures which is related to drug prescribing patterns.

Since our aims were to investigate both drug prescribing patterns and consequent OPE, we deemed interviewing patients to be the most informative and insightful method of investigation. However, while the household perspective enabled us to collect information on both purchasing and prescribing patterns, recall bias cannot be ruled out, in particular with regard to drug prescribing. We aimed to minimize this risk by using a short timeframe between FD consultations and interviews and, wherever possible, by making use of available prescriptions.

### Drug prescription

In Tajikistan, doctors at PHC level seem to overprescribe drugs, in view of the number of drugs prescribed concomitantly and the high level of polypharmacy [11]. It can be explained by the low official salary FD earn from the government, estimated to range between US$ 123 and US$ 153 per month in 2013 [5] which is not enough to cover essential needs. As a consequence, doctors often rely on informal payments and in-kind contributions from patients. Expenditures for drugs represented 2.8 % of governmental health budget in 2013 [2] so that pharmaceuticals are mainly financed by patients through informal OPE (both at hospital and primary care level). Such conditions do not favour rational prescribing as the prescription of a high number of drugs can represent a complementary income source to doctors and pharmacists.

In most cases, patients reported trusting their doctor’s advice and there was little self-procurement of drugs without prescriptions. However, the results also highlight geographical disparities between districts of republican subordination (RRS) and Khatlon region (*p* = 0.05). Reasons could be that doctors and pharmacists are more widely influenced by the pharmaceutical industry in the RRS, being geographically close to the capital city Dushanbe. Another hypothesis could be that the RRS region hosts a higher proportion of well-off households than the Khatlon region with patients having a higher purchasing power, utilizing health services more frequently. In support of the latter hypothesis is earlier research that found that richer groups of the population utilize health services more than poorer groups. In the 2011 Panorama Household Survey, utilization of outpatient care by the richest quintile was almost twice as high as by the poorest quintile and utilization of inpatient care was almost three times higher among the richest quintile compared with the poorest quintile [7].

### Characteristics of the drugs prescribed

Vitamins were issued in 61.0 % of cases, an extremely high number that cannot be explained by the prevalence of diseases caused by micronutrient deficiencies. The characteristics of the drugs prescribed were similar to those found in Uzbekistan [8]. Vitamin injections or intravenous rehydration were also found to be commonly prescribed in a study on TB patients in Tajikistan [12]. In addition to being of questionable clinical value and inducing an unnecessary financial burden on vulnerable households when oral drugs could be a cheaper alternative, injections are associated with the risk of infections and thus potentially harmful to patients. This is in line with research in other former Soviet countries, including Tajikistan, which found that clinical practice is still far from internationally accepted evidence and that many harmful practices persist [13].

Our findings on the excessive use of antibiotics are in line with previous studies that have shown the inappropriate use of antibiotics in Tajikistan [14]. The high prescription of antibiotics results in increased drug expense, exacerbates drug resistance, and presents a danger to the health of the population which could ultimately undermine trust in the health sector. A recent study of low- and middle-income countries found that patient co-payments in the public sector were associated with an increased use of antibiotics, potentially due to supply-side incentives and underdeveloped mechanisms for quality assurance [15]. This corroborates well established evidence on the effects of fee-for-services arrangements, which tend to lead to excessive investigations, treatment and prescription [16]. An added challenge in countries such as the former Soviet ones is the existence of informal, under-the-counter payments which not only lead to perversive incentives for physicians, but also errode transparency and accountability more generally [17].

### Drug procurement

Private drug-selling retail points have grown across the country over the past decade which substantially improved drug availability in Tajikistan. However, the predominant role of the private sector and the underdeveloped regulatory environment have led to an underuse of generic drugs over the delivery of brand names. Already in 2005, the inclusion of 139 brand names in the Tajikistan Essential Drug List was a source of concern for the WHO, suggesting that the Tajik government had not embraced the promotion of generic drugs, and instead allowed the entry of expensive drugs into the market which the country could not afford [18]. Although the situation has improved since then, the lack of evidence-based guidelines, the insufficient education of doctors and pharmacists, and the pressure from the pharmaceutical industry all result in irrational prescribing practices. The prescription of brand names instead of International Nonproprietary Names (INN) for pharmaceutical substances means that patients purchase expensive drugs instead of generics, ultimately impairing access of poor households to health care.

### Expenditures for drugs

Despite ongoing health reforms aiming to improve coverage and financial protection, OPE remain substantial. In our study, expenditures on drugs represented the biggest financial burden for patients accessing a primary care facility. One of the few studies published on the burden of this expenditure to household income in Tajikistan indicates, using 2003 data, that around 5 % of the household income is allocated to health [19]. Additional evidence from 2011 indicates that as a consequence of the substantial reliance on OPE, 26.7 % of households in the lowest consumption quintile are at risk of catastrophic health expenditure by spending over 40 % of their non-subsistence spending on health [20].

With the continuing predominance of OPE to finance health services and without a social risk protection scheme, Tajik households are put at risk of impoverishment and of catastrophic health expenditure in case of major illness episodes. These results suggest that the health reforms being implemented in Tajikistan aiming to offer free primary care services, reduce OPE and strengthen the role of family medicine, including through the Basic Benefit Package, do not yet yield the expected benefits.

## Conclusion

In conclusion, analysis of the survey we conducted in 2014 in rural and semi-urban Tajikistan showed a high rate of drug prescription, an irrational use of antibiotics and vitamins and the common use of injections to administer medicines at the PHC level. Expenditures for drugs represented more than three-quarters of the total amount paid in the course of a visit to a PHC provider. It resulted that a third of the interviewees who did not obtain the drugs prescribed explained it to their inability to pay.

Efforts to develop, disseminate and ensure implementation of national protocols and guidelines for PHC providers need to be strengthened to improve rational and evidence-based prescribing. This includes doctors using INN when issuing a prescription to allow the dissemination of generic drugs. The resulting private and public resource savings could be allocated to other parts of the health system.

On the demand side, it is crucial to raise the population’s awareness of generic drugs so as not to impair their trust in the behaviour of physicians and pharmacists. Communication should also be encouraged to explain the rational use of antibiotics, safer and cheaper ways of administrating medications and the limited clinical value of vitamin supplements (except for vitamin deficiencies).

In the Tajik context where OPE are important, drugs represent an important income source for various health system players, principally pharmacists incentivized to deliver drugs without prescription to increase their salary. Such situations do not favour rational prescribing nor efficient service delivery, and are potentially inappropriate or even harmful for patients. Patients’ and households’ interests are put at risk, especially the more vulnerable segments of the population.

## Additional file

Additional file 1: Figure S1. Questionnaire of the 2014 study. (DOCX 28 kb)
