# Supplementary material for: Drug prescribing patterns at primary health care level and related out-of-pocket expenditures in Tajikistan
Source: BMC Health Serv Res. 2016 Oct 6;16:556. doi: 10.1186/s12913-016-1799-2 (PMC5053171; doi:10.1186/s12913-016-1799-2)
Supplement: Additional file 1: Figure S1. — Questionnaire of the 2014 study. (DOCX 28 kb) [file 12913_2016_1799_MOESM1_ESM.docx]

**Figure 1: Questionnaire of the 2014 study**

| **N** | **Question** | **Code** |
| --- | --- | --- |
|  |  | |
|  | **PART I - IDENTIFICATION** | |
| 1 | Patient Identification Number |  |
| 2 | Patient consultation date | Day/month |
| 3 | Reason for consultation | 1= Respiratory  2= Digestive/ gastrointestinal  3= Cardiovascular including hypertension  4= Diarrhoea  5= Pregnancy  6= Genitourinary  7= Injuries  8= Skin disease  9= Diabetes  10= Anaemia  11= Other |
| 4 | Interviewer Code |  |
| 5 | Rayon Code | 1 = Dangara  2 = Varsob  3 = Tursunzade  4 = Shakrinav  5 = Vose  6 = Khamadoni  7 = Rudaki  8 = Faizabad |
| 6 | Rural Health Centre Code |  |
|  |  | |
|  | **PART II – PATIENT EXPERIENCE** | |
| **Section 1 - Experience with facility use** | | |
| 7 | Is the distance from your home to the health centre acceptable? | 1= Yes, it's not a long distance and it's easy for me to come here  2= The centre is not close, but it's ok for me to come here  3= No, the centre is far away and it's difficult for me to come here |
| 8 | How far away is the health centre from your home? | 1= Less than 15 minutes  2= 15-29 minutes  3= 30-59 minutes  4= 60 minutes or more |
| 9 | At the health centre, were you provided with information/leaflets that allows you to understand common diseases in your region, their treatment and prevention? | 1 = Yes  2 = No  3 = Can’t remember |
| 10 | Do you regularly receive the health messages from the community groups, existing in your village and then discuss them among communities? | 1= Yes, I regularly receive  2= Sometimes I receive  3= No, I don't receive  4= I'm not aware of such activities |
|  | | |
| **Section 2 – Patient satisfaction** | | |
| 11 | Did the doctor examine your body as you expected he/she to do? | 1= Yes, he/she examined me  2= He/she did not do all the exams I though he/she would do  3= He/she did more than I thought was necessary  4= No, he/she did not examined me at all  5= I do not remember |
| 12 | During your last visit, were you given adequate information on your health condition? | 1 = Yes, very clear information/ explanations were given  2 = He /she could have explained in a better way  3 = No, not at all  4 = I did not need information |
| 13 | During your last visit, were you given adequate information on your treatment? | 1 = Yes, very clear information/ explanations were given  2 = He /she could have explained in a better way  3 = No, not at all  4 = I did not need information |
| 14 | Overall, how satisfied are you with the care you received by the Family Doctor during this past visit?  *Instruction: Present sheet with possible answers (read out-loud if patient wishes)* | 1 = Very satisfied  2 = Satisfied  3 = Neither satisfied nor dissatisfied  4 = Dissatisfied  5 = Very Dissatisfied |
|  | | |
| **Section 3 – Main outcomes of interest** | | |
| 15 | Did the doctor send you to a specialist (polyclinic/hospital/specialized centres, dispensary, etc)? | 1 = Yes  2 = No  3 = Can’t remember |
| 16 | Did you give any money to the Family Doctor or to the Family Nurse? | 1 = Yes  2 = No |
| 17 | How much money did you give to the Family Doctor or to the Family Nurse? | Tajik Somoni  999 = Don’t know |
| 18 | Did you give any non-monetary gifts to the Family Doctor or to the Family Nurse? What was the approximate value of the gift? | Value in Tajik Somoni  999 = Don’t know |
| 19 | What resources did you use? Where did the money come from? | 1= Savings  2= Borrowing in Tajikistan  3= Borrowing from relatives abroad  4= Credit  5= Sell farm products(animals, produce)  6= Sell valuables |
| 20 | How much money did you spend traveling to consult your family doctor? | Value in Tajik Somoni  999 = Don’t know |
| 21 | Did the Family Doctor prescribe drugs for you during the consultation? | 1 = Yes  2 = No – go to question 33 |
|  | | |
| **Section 4 - Drug Purchase** | | |
| 22 | How many drugs were prescribed? | 1=1  2=2  3=3  4=4  5=5  6=6  7=7  8= >=8 |
| 23 | How many drugs were bought? | 1= 1  2=2  3=3  4=4  5=5  6=6  7=7  8= >=8 |
| 24 | Among the drugs bought, are there any transfusion (intravenous injection)? | 1 = Yes  2 = No  3 = Don’t know |
| 25 | Among the drugs bought, are there any non-intravenous injection? | 1 = Yes  2 = No  3 = Don’t know |
| 26 | Among the drugs bought, are there any antibiotics? | 1 = Yes  2 = No  3 = Don’t know |
| 27 | Among the drugs bought, are there any vitamins? | 1 = Yes  2 = No  3 = Don’t know |
| 28a. | Considering the first drug, where did you buy it? | 1= Pharmacy  2= Market  3= Family Doctor  4= Hospital  5= Other |
| 28b. | Considering the second drug, where did you buy it? | 1= Pharmacy  2= Market  3= Family Doctor  4= Hospital  5= Other |
| 28c. | Considering the third drug, where did you buy it? | 1= Pharmacy  2= Market  3= Family Doctor  4= Hospital  5= Other |
| 28d. | Considering the fourth drug, where did you buy it? | 1= Pharmacy  2= Market  3= Family Doctor  4= Hospital  5= Other |
| 28e. | Considering the fifth drug, where did you buy it? | 1= Pharmacy  2= Market  3= Family Doctor  4= Hospital  5= Other |
| 28 f. | Considering the sixth drug, where did you buy it? | 1= Pharmacy  2= Market  3= Family Doctor  4= Hospital  5= Other |
| 28g. | Considering the seventh drug, where did you buy it? | 1= Pharmacy  2= Market  3= Family Doctor  4= Hospital  5= Other |
| 28h. | Considering the eighth drug, where did you buy it? | 1= Pharmacy  2= Market  3= Family Doctor  4= Hospital  5= Other |
| 29 | In total, how much money did you spend on these drugs? | Tajik Somoni  999 = Don’t know |
| 30 | How much money did you spend traveling to obtain these drugs? | Tajik Somoni  999 = Don’t know |
| 31 | What was the main reason why you did not obtain (all) the prescription drugs? (if number of drugs bought<number of drugs prescribed) | 1 = No pharmacy near by  2 = No money  3 = Pharmacy did not have drug in stock  4 = Did not feel I needed this drug  5 = Other |
| 32 | Did you use any other non-prescribed drug? | 1= Yes, from pharmacy  2= Yes, available in my house  3= Yes, other, e.g.  4= No |
| 33 | Did you use any supplementary treatment? | 1= Yes, e.g  2= No |
|  |  | |
|  | **PART III – DEMOGRAPHICS** | |
| 34 | Are you male or female? | 1 = Male  2 = Female |
| 35 | What is your age? | In completed years |
| 36 | What is your main source of income household? | 1= Private business  2= Salary  3= Pension or social aid  4= Farming & livestock  5= Other |
| 37 | How many years of education have you undertaken? | Number of years |
| 38 | In the past 12 months how many times have you visited a doctor for a consultation on a health related issue? | Number of visits |
| 39 | How would you rate your current health status? | 1 = Very good (I rarely need to go to the doctor)  2 = OK (Sometimes I need to go and talk to the doctor)  3 = Poor (I very often/constantly need to see the doctor)  4 = Very poor (I can do almost nothing without being helped by a health professional) |
| 40 | How many people regularly live in the same house with you? |  |
| 41 | Number of rooms in the house |  |
| 42 | Main source of drinking water | 1 = River, ditch or aryk  2 = Well or spring  3 = Public tap  4 = Tap at home  5 = Delivered from other place  6 = Mineral water |
| 43 | What type of toilet facility is being used by your household? | 1 = Hole  2 = Flushing toilet  3 = Public toilet  4 = No toilet |
| 44 | Do you have electricity at your house? | 1 = Yes  2 = No |
| 45 | What material is the floor of the house | 1 = Wooden  2 = Concrete with cover  3 = Loam (just earth)  4 = Other |
| 46 | What material are the walls of the house | 1 = Clay (loyi)  2 = Baked brick  3 = Cement (beton, stone)  4 = Other |
| 47 | On average how many times in one month do you typically consume meat (during the time when you are healthy)? | 1 = Zero  2 = Once per month  3 = 2-5 times per month  4 = 6-10 times per month  5 = Over ten times per month  6 = Every day  7 = Vegetarian |
|  |  | |
|  | **PART IV - OWNERSHIP**: Does your household own ..... ? And if so, how many? | |
| 48 | A bicycle? | 1= Yes  2= No |
| 49 | A motorbike and/or scooter? | 1= Yes  2= No |
| 50 | A car? | 1= Yes  2= No |
| 51 | A truck? | 1= Yes  2= No |
| 52 | Animal drawn cart? | 1= Yes  2= No |
| 53 | Agricultural land? | 1= Yes  2= No |
| 54 | Farm animals (any livestock, herds, other farm animals, beehives or poultry)? | 1= Yes  2= No |
| 55 | A refrigerator? | 1= Yes  2= No |
| 56 | An indoor heater/ burzhuika? | 1= Yes  2= No |
| 57 | A stock of wood or other fuel? | 1= Yes  2= No |
| 58 | A television? | 1= Yes  2= No |
| 59 | A DVD player? | 1= Yes  2= No |
| 60 | A satellite antenna/ dish? | 1= Yes  2= No |
| 61 | A computer? | 1= Yes  2= No |
| 62 | A phone (land and/or mobile)? | 1= Yes  2= No |
| 63 | A watch? | 1= Yes  2= No |
| 64 | An electric oven? | 1= Yes  2= No |
